# Supplementary material for: Distinct early-life mechanisms of quantity discrimination in domestic chicks
Source: PLoS One. 2026 May 22;21(5):e0349870. doi: 10.1371/journal.pone.0349870 (PMC13196943; doi:10.1371/journal.pone.0349870)
Supplement: Attachment — Submitted filename: PlosOne_Loconsole_review.docx [file pone.0349870.s002.docx]

***Plos One Peer review* - Loconsole et al., 2026**

In this manuscript, the authors replicate two experiments on numerosity in young chicks (choice of the larger group after imprinting, and preference for the “prime” number following habituation to even numerosities), with the aim of standardizing the setup and experimental conditions. While this objective is valuable and worth pursuing, several issues limit the strength of the claims.

**1) Lack of true standardization across experiments**

The stated goal of standardizing conditions across the two experiments is not fully achieved. Although the authors employ a common physical setup and the same test numerosities (5 vs 9), the stimuli themselves differ substantially.

In Experiment 1, physical red cylinders are used, whereas in Experiment 2 stimuli are digitally projected on a screen and vary in both color and shape. This discrepancy represents a significant limitation for two main reasons: 1) It weakens the claim of methodological standardization across experiments; 2) It limits the degree of stimulus control, particularly in Experiment 1 (see point 2).

Designing Experiment 1 using digitally controlled stimuli, as in Experiment 2, would have allowed for tighter control of continuous variables and improved comparability between experiments. It is unclear why this issue is neither acknowledged nor discussed.

**2) Insufficient control and discussion of non-numerical continuous variables**

Despite the title emphasizing numerical discrimination, the manuscript does not adequately address continuous variables that chicks might use instead of number (e.g., total area, perimeter, density, spatial arrangement, convex hull). This omission substantially weakens the interpretation of the results: without explicit control or analysis of these factors, it remains unclear whether the observed effects genuinely reflect numerical processing.

This concern is particularly relevant for Experiment 1, where the use of physical objects makes precise control of continuous variables more difficult. This further supports the argument that digital stimuli would have been more appropriate.

Regarding Experiment 2, although digital stimuli were used, there is no discussion of their geometrical properties or whether continuous variables covaried with number and potentially contributed to the results. Such analyses are essential.

A further issue concerns the habituation phase. During habituation, chicks were exposed to numerosities 4, 6, 10, and 12, each apparently associated with a specific color and shape (the manuscript should clearly specify which color/shape corresponded to each numerosity). Importantly 4 is smaller than both test numerosities (5 and 9); 6 lies between them, 10 and 12 are larger than both.

It would be important to test whether choices at test are modulated by the specific color/shape previously associated with different numerical magnitudes. An analysis stratified by habituation category (smaller, intermediate, larger than test values) could clarify whether the observed effects depend on prior magnitude exposure rather than numerical properties per se.

**3) Theoretical framework of “prime number” choice**

There are conceptual concerns regarding the interpretation of results in terms of prime number detection.

First, the test condition (5 vs 9) alone is insufficient to support a strong claim about prime number preference; additional testing pairs would be necessary to substantiate such a conclusion.

Second, although the rationale is based on Loconsole et al., 2021, the theoretical background is not adequately explained in the current manuscript. The explanation is brief and unclear. In the earlier work, the emphasis appears to be on symmetry properties and grouping mechanisms rather than on prime numbers per se. The novelty or asymmetry of grouping patterns seems central to the interpretation.

The current manuscript risks overstating “prime number detection” without sufficiently grounding it in the symmetry/grouping framework. This conceptual framing should be clarified and expanded.

Moreover, if the proposed mechanism relies on symmetry properties, then the spatial arrangement and configuration of elements become critical factors. A deeper analysis of stimulus configuration and grouping structure would strengthen the interpretation.

***Overall assessment and recommendations***

Substantial revision is necessary. In particular:

- Experiment 1 should ideally be redesigned using digitally controlled stimuli to match Experiment 2 and to allow systematic control of continuous variables (e.g., varying area, perimeter, density, convex hull during imprinting to prevent their use as informative cues).
- Experiment 2 should include analyses assessing the contribution of continuous variables that covary with number.
- Test performance should be analyzed as a function of the color/shape associated with specific habituation numerosities.
- The discussion should be substantially revised to address these methodological issues and to more accurately frame the concept of prime number detection within the broader context of symmetry and grouping mechanisms.

Without these adjustments, the conclusions regarding numerical discrimination and prime number preference remain insufficiently supported.
