# Supplementary material for: Distinct early-life mechanisms of quantity discrimination in domestic chicks
Source: PLoS One. 2026 May 22;21(5):e0349870. doi: 10.1371/journal.pone.0349870 (PMC13196943; doi:10.1371/journal.pone.0349870)
Supplement: Attachment — Submitted filename: Response_to_review_Loconsole.docx [file pone.0349870.s004.docx]

**Reviewer #1:**

In this manuscript, the authors test whether two apparently divergent findings in the literature on early numerical cognition in domestic chicks can be replicated under closely aligned methodological conditions. One line of research has shown that chicks imprinted on a set of identical objects preferentially approach the larger numerosity at test. Another line has demonstrated that chicks habituated to heterogeneous even-numbered sets display longer inspection of prime-numbered sets, suggesting reliance on perceptual grouping rather than magnitude per se. To directly compare these phenomena, the authors tested two independent groups of 24-hour-old chicks using the same numerical comparison (5 vs. 9), the same arena, and the same dependent measure (time spent in proximity to each set). They report a significant interaction between numerosity and pre-test procedure: imprinted chicks prefer the larger set (9), whereas habituated chicks spend more time near the prime set (5), replicating both previously reported effects under aligned conditions.
This is a well-designed, methodologically careful, and clearly written study. The statistical approach is appropriate and transparent, inter-rater reliability is strong, and the attempt to equate key methodological parameters across paradigms is particularly commendable. Importantly, the manuscript contributes to the literature by conducting a comparative replication of two apparently opposed effects, showing that both are robust and replicable when tested under similar experimental constraints. This comparative approach substantially strengthens confidence in the existence of multiple early-life mechanisms supporting numerical discrimination in chicks. I therefore strongly recommend publication, after minor revisions aimed at strengthening the theoretical framing and clarifying certain conceptual distinctions.

We are grateful to the Reviewer for their encouraging and constructive comments on our manuscript. We appreciate the time and care taken to evaluate our work. We have carefully considered all comments and suggestions and have revised the manuscript accordingly. Our detailed responses to each point are provided below.

Detailed comments

1. Expand the discussion on why the two procedures elicit different mechanisms. While the authors successfully demonstrate that imprinting leads to a preference for the larger familiar set and habituation leads to a preference for the prime set, the discussion would benefit from a slightly more explicit consideration of why these procedures might differentially engage distinct cognitive systems.

We thank the reviewer for this helpful suggestion. Following this comment, we have expanded the Discussion to more explicitly consider why imprinting and habituation procedures may engage distinct cognitive mechanisms.

Please see lines: 298 – 317

*From an evolutionary perspective, this tendency may be advantageous, as recognising and approaching a larger number of social companions can support brood cohesion, reduce predation risk through aggregation, and improve opportunities for foraging and information transfer within the group. Numerical cognition thus plays a key role in group dynamics and constitutes a foundation for ecological success in many social species, including the domestic chicken [32]. Notably, previous work has also shown that when chicks are reared with objects differing in colour, size, and shape, and are subsequently tested with completely novel objects, they express a preference toward the familiar, rather than larger, numerosity [14]. This evidence highlights how stimulus composition and the representational format available during early experience can modulate the outcome of imprinting, reinforcing the view that early numerical behaviour depends critically on how perceptual and motivational systems are structured by experience.*

*For what concerns habituation, exposure to visually variable stimuli seems to elicit a novelty-based strategy grounded in perceptual grouping. When imprinting-related social mechanisms are not engaged, chicks may rely more strongly on exploratory processes that favour slight novelty. Such a tendency is also adaptive in natural environments, as it enables animals to detect and respond to subtle changes in their surroundings while maintaining contact with familiar and safe stimuli. Preferring slight, rather than extreme, novelty may therefore represent a balanced strategy that promotes learning and environmental updating without abandoning potentially protective cues [33,34].*

2. Situate results within imprinting studies using heterogeneous stimuli. The discussion would benefit from acknowledging that not all imprinting paradigms lead to a preference for the larger numerosity. Previous work has shown that when chicks are imprinted on heterogeneous objects, they may exhibit a preference for the familiar numerosity, rather than the larger one.
This literature is highly relevant here because it suggests that imprinting does not uniformly trigger a magnitude-based strategy; instead, stimulus composition and representational format can modulate the outcome. Integrating these findings would reinforce the manuscript’s broader claim that early numerical behavior depends critically on how experience structures perceptual and motivational systems.
We thank the Reviewer for pointing out this relevant consideration. We have clarified in the introduction and in the discussion that a preference for the larger set following imprinting is usually observed after rearing with identical objects.

Please see lines: 47– 48 and 296 – 298

*A well-established finding in the literature is that chicks can discriminate between groups of identical objects with which they were reared for several hours or days shortly after hatching.*

*Imprinting to identical objects appears to trigger processes related to social affiliation and re-joining, leading chicks to favour the larger set of familiar objects.*

Additionally, we have implemented this insightful suggestion proposed by the Reviewer in our discussion.

Please see lines: 303 – 309

*Notably, previous work has also shown that when chicks are reared with objects differing in colour, size, and shape, and are subsequently tested with completely novel objects, they express a preference toward the familiar, rather than larger, numerosity [14]. This evidence highlights how stimulus composition and the representational format available during early experience can modulate the outcome of imprinting, reinforcing the view that early numerical behaviour depends critically on how perceptual and motivational systems are structured by experience.*

In this regard, it is important to note that the stimuli used during habituation cannot be straightforwardly classified as either homogeneous or heterogeneous in terms of shape. Within each individual set, elements were homogeneous (all items shared the same shape and colour), but every 10 seconds a new set composed of different shapes and numerosities was presented, resulting in an overall visually heterogeneous experience across the habituation phase.

We agree with the Reviewer and we have adopted the more correct terminology of “visually variable stimuli” throughout the manuscript.

3. Clarify the conceptual distinction between imprinting and habituation. The manuscript treats imprinting and habituation as distinct pre-test procedures, which is experimentally accurate. However, from a developmental perspective in precocial species such as the domestic chick, this distinction may not be entirely straightforward. We know that imprinting learning may begin after relatively short exposure durations and that, especially short imprinting periods can give rise to novelty preferences (see the old works of Patrik Bateson on this regard). Given this, it would be useful for the authors to clarify on what theoretical basis they consider the habituation condition to engage mechanisms fundamentally distinct from imprinting learning

We thank the Reviewer for raising this important conceptual point. In addition to the modifications introduced following the Reviewer’s first comment, we have now revised the Introduction to further clarify why the two exposure conditions were expected to favour different learning processes.

Please see lines: 56 – 60 and 77 – 81

*When imprinting stimuli possess stable features that animals can reliably detect, these features are integrated into a single mental representation, and the object as a whole is learned as an individual. As a consequence, when given a choice, chicks tend to orient towards the larger group of such familiar individuals.*

*Exposure to continuously varying stimuli prevents chicks from learning the individual features of each set and instead promotes the extraction of a more general regularity shared across experiences. In this scenario, chicks are less likely to respond to the larger set, as observed with imprinted objects, and are more likely to respond to the abstract rule or pattern that characterises the experienced stimuli.*

**Reviewer #2**

In this manuscript, the authors replicate two experiments on numerosity in young chicks (choice of the larger group after imprinting, and preference for the “prime” number following habituation to even numerosities), with the aim of standardizing the setup and experimental conditions. While this objective is valuable and worth pursuing, several issues limit the strength of the claims.

**1) Lack of true standardization across experiments**

The stated goal of standardizing conditions across the two experiments is not fully achieved. Although the authors employ a common physical setup and the same test numerosities (5 vs 9), the stimuli themselves differ substantially.

In Experiment 1, physical red cylinders are used, whereas in Experiment 2 stimuli are digitally projected on a screen and vary in both color and shape. This discrepancy represents a significant limitation for two main reasons: 1) It weakens the claim of methodological standardization across experiments; 2) It limits the degree of stimulus control, particularly in Experiment 1 (see point 2).

Designing Experiment 1 using digitally controlled stimuli, as in Experiment 2, would have allowed for tighter control of continuous variables and improved comparability between experiments. It is unclear why this issue is neither acknowledged nor discussed.

We agree with the Reviewer that the fact that the stimuli differed between the two experiments

limits the degree to which the procedures can be considered fully standardised. However, it is important to clarify that in both Exp. 1 and Exp. 2 the stimuli were not controlled for continuous variables. As such, continuous quantity information was available to the chicks in both cases, irrespective of whether the stimuli were presented as 3D objects or as 2D projections. The main reason for employing different stimuli was to maximise the likelihood that the two procedures would engage the intended mechanisms.

In Exp. 1, we employed physical objects because imprinting responses are generally stronger when stimuli are more salient. Although imprinting can occur with two-dimensional stimuli, three-dimensional objects are known to elicit more robust affiliative responses. Given that our goal was to measure magnitude-based preferences at an earlier age than typically tested, we opted to enhance stimulus saliency to facilitate the expression of imprinting behaviour.

In contrast, the habituation procedure used in Exp. 2 required the rapid sequential presentation of multiple sets differing in numerosity and visual properties. Under these conditions, presenting 3D stimuli was not methodologically feasible. The use of digitally presented stimuli therefore allowed us to implement the rapid succession of visually varying sets required to promote habituation and to minimise the likelihood of triggering imprinting related processes.

We nonetheless agree with the Reviewer that the use of different stimulus formats represents an aspect that deserves consideration. In the revised manuscript, we have clarified the rationale behind the choice of stimuli in the manuscript and added a discussion noting that future studies could explore the extent to which similar results are obtained when the two procedures are implemented using more comparable stimulus formats.

Please see lines: 254 – 263

*Importantly, the two experiments were designed to minimise procedural differences, allowing a more direct comparison. Chicks were tested at the same age, in the same arena, with the same numerical discrimination, and the same behavioural measure was recorded at test. Nevertheless, we retained a difference in the stimuli employed: Exp. 1 used 3D physical objects, whereas Exp. 2 used 2D digital projections. This choice was motivated by the need to increase objects saliency and strengthen imprinting responses in Exp. 1, while allowing a rapid presentation of varying two-dimensional stimuli in Exp. 2, thereby maximising the likelihood that each procedure engaged the targeted cognitive system. Although our approach proved effective, future studies could investigate more closely matched stimulus formats to further evaluate the generalisability of these effects.*

**2) Insufficient control and discussion of non-numerical continuous variables**

Despite the title emphasizing numerical discrimination, the manuscript does not adequately address continuous variables that chicks might use instead of number (e.g., total area, perimeter, density, spatial arrangement, convex hull). This omission substantially weakens the interpretation of the results: without explicit control or analysis of these factors, it remains unclear whether the observed effects genuinely reflect numerical processing.

This concern is particularly relevant for Experiment 1, where the use of physical objects makes precise control of continuous variables more difficult. This further supports the argument that digital stimuli would have been more appropriate.

Regarding Experiment 2, although digital stimuli were used, there is no discussion of their geometrical properties or whether continuous variables covaried with number and potentially contributed to the results. Such analyses are essential.

A further issue concerns the habituation phase. During habituation, chicks were exposed to numerosities 4, 6, 10, and 12, each apparently associated with a specific color and shape (the manuscript should clearly specify which color/shape corresponded to each numerosity). Importantly 4 is smaller than both test numerosities (5 and 9); 6 lies between them, 10 and 12 are larger than both.

It would be important to test whether choices at test are modulated by the specific color/shape previously associated with different numerical magnitudes. An analysis stratified by habituation category (smaller, intermediate, larger than test values) could clarify whether the observed effects depend on prior magnitude exposure rather than numerical properties per se.

We thank the Reviewer for raising this point and for the opportunity to clarify the rationale of our design.

In both Experiments, continuous variables that covary with numerosity were not strictly controlled. This choice was intentional and related to the specific aim of the study. Our goal was not to determine whether chicks rely on abstract numerosity as opposed to continuous magnitude cues, but rather to examine whether different early-life procedures, imprinting and habituation, would lead to distinct behavioural outcomes when chicks were presented with sets differing in quantity. In this context, “smaller” and “larger” should be intended in terms of overall magnitude rather than necessarily abstract number. For this reason, we intentionally allowed the full range of magnitude-related information to remain available to the chicks. This approach aimed to capture a more naturalistic processing of quantitative stimuli, rather than isolating abstract numerical information.

Importantly, even if chicks relied on continuous variables correlated with quantity, this alone would not explain the dissociation observed between the two experiments. After imprinting, chicks preferred the larger set, whereas following habituation they showed a preference consistent with novelty, which in our test corresponded to the smaller set. This divergence supports the interpretation that different mechanisms were engaged by the two procedures.

We have added a consideration on this aspect in the discussion of the revised manuscript. Please see lines: 283 – 291

*In both experiments, continuous variables covarying with numerosity were not strictly controlled, in order to more closely resemble natural conditions in which multiple magnitude-related cues remain available. The dissociation observed between experiments, with imprinting leading to a preference for the larger set and habituation eliciting a novelty-related response, suggests that distinct cognitive mechanisms were engaged by the two procedures regardless of the specific quantitative cue guiding the discrimination. It remains an open question whether constraining chicks to rely specifically on abstract numerosity, by controlling for continuous variables that typically covary with it, would lead to a different strategy or behavioural outcome.*

Regarding Exp. 2, there was no fixed correspondence between numerosity, colour, and shape, but each presented set consisted of a random combination of these three features. This rules out the possibility that chicks were responding to one dimension only, as they never correlated in a consistent manner.

We have now clarified this aspect in the text. Please see lines: 164 – 166

*Each stimulus consisted in a random combination of shape (i.e., triangles, rectangles, or circles), colour (i.e., yellow, red, blue, or green) and numerosity (always even, i.e., 4, 6, 8, 10).*

**3) Theoretical framework of “prime number” choice**

There are conceptual concerns regarding the interpretation of results in terms of prime number detection.

First, the test condition (5 vs 9) alone is insufficient to support a strong claim about prime number preference; additional testing pairs would be necessary to substantiate such a conclusion.

Second, although the rationale is based on Loconsole et al., 2021, the theoretical background is not adequately explained in the current manuscript. The explanation is brief and unclear. In the earlier work, the emphasis appears to be on symmetry properties and grouping mechanisms rather than on prime numbers per se. The novelty or asymmetry of grouping patterns seems central to the interpretation.

The current manuscript risks overstating “prime number detection” without sufficiently grounding it in the symmetry/grouping framework. This conceptual framing should be clarified and expanded.

We agree that the test pair used in the present study, 5 vs 9, would not be sufficient on its own to support a general claim about responses to prime numerosities. However, as the Reviewer rightfully noticed, the rationale for this comparison derives from previous work in which multiple numerical pairs were tested using the same theoretical framework. In Loconsole et al. (2021), chicks were tested with several pairs of numerosities, including 7vs9, 9vs11, and 13vs15, allowing the authors to show that chicks consistently responded to a specific perceptual property associated with prime numerosities.

Additionally, we would like to emphasise that our interpretation does not imply that chicks detect prime numbers in a formal mathematical sense. Rather, the claim concerns a perceptual property that is characteristic of prime numerosities. Previous work suggests that chicks spontaneously rely on grouping strategies when processing sets of objects and prime numerosities stand out because they resist symmetrical grouping, leading to perceptually irregular patterns. Thus, the preference observed in these studies is to be interpreted as a response to the asymmetry or novelty generated by non factorisable groupings, rather than as evidence of explicit prime number representation.

We have now clarified this point in the manuscript to make explicit that the present experiment builds on that broader empirical framework and to avoid possible misunderstandings on the theoretical basis for our claim.

Please see lines: 61 – 81

*More recent findings, however, indicate that chicks can also solve numerical discriminations using a non-mathematical strategy, and in particular they could respond to a perceptual property that is characteristic of prime numerosities. Sets containing a prime number of elements cannot be partitioned into smaller equal-sized subsets. Any attempt to group such sets into equal parts necessarily results in at least one subset differing in numerosity, producing a perceptually imbalanced configuration. In contrast, non-prime numerosities can be decomposed into equal groups, generating more symmetrical and perceptually balanced arrangements. In a first study, newly hatched chicks were habituated for one hour to 360 sets of bidimensional objects varying in shape, colour, and numerosity [15]. They were then tested for their preference between two novel sets: one of a novel prime numerosity and one of a novel non-prime odd numerosity (7vs.9, 9vs.11, or 13vs.15). Results showed that chicks spent more time inspecting the set with the prime numerosity, regardless of whether it was the smaller or larger of the pair. Under the proposed framework, chicks were able to discriminate prime and non-prime numerosities because the first resist symmetrical grouping, leading to perceptually irregular patterns. The preference observed in these paradigms is therefore interpreted as a response to the asymmetry or novelty generated by non factorisable groupings. Exposure to continuously varying stimuli prevents chicks from learning the individual features of each set and instead promotes the extraction of a more general regularity shared across experiences. In this scenario, chicks are less likely to respond to the larger set, as observed with imprinted objects, and are more likely to respond to the abstract rule or pattern that characterises the experienced stimuli.*

Moreover, if the proposed mechanism relies on symmetry properties, then the spatial arrangement and configuration of elements become critical factors. A deeper analysis of stimulus configuration and grouping structure would strengthen the interpretation.

We thank the Reviewer for raising this point that allows us to better clarify our methodology. In both Exp. 1 and Exp. 2 the spatial configuration of the stimuli was random, and therefore non-informative for the discrimination.

Please see lines: 166 – 168

*Each element had an approximate area of 36px, whilst the spatial arrangement was random within a white area (336px) in the middle of the screen.*

***Overall assessment and recommendations***

Substantial revision is necessary. In particular:

- Experiment 1 should ideally be redesigned using digitally controlled stimuli to match Experiment 2 and to allow systematic control of continuous variables (e.g., varying area, perimeter, density, convex hull during imprinting to prevent their use as informative cues).
- Experiment 2 should include analyses assessing the contribution of continuous variables that covary with number.
- Test performance should be analyzed as a function of the color/shape associated with specific habituation numerosities.
- The discussion should be substantially revised to address these methodological issues and to more accurately frame the concept of prime number detection within the broader context of symmetry and grouping mechanisms.

Without these adjustments, the conclusions regarding numerical discrimination and prime number preference remain insufficiently supported.

We thank the Reviewer for the careful evaluation of our manuscript and for the constructive suggestions provided. We appreciate the opportunity to clarify several methodological and conceptual aspects of the study.

In the revised manuscript, we have addressed the points raised by providing additional clarification of our methodological choices, expanding the discussion of continuous variables and their role in the present design, and further elaborating the theoretical framework underlying our interpretation of the results. In particular, we have clarified the rationale for the use of different stimulus formats across the two experiments, specified the structure of the habituation stimuli, and reframed the interpretation of the results in terms of perceptual grouping and symmetry properties rather than prime number detection in a formal mathematical sense.

We believe these revisions improve the clarity of the manuscript and more accurately position our findings within the relevant theoretical context.
